# Assessing the cadmium content of cacao crops in Arauca, Colombia

**DOI:** 10.1007/s10661-024-12539-9

**Published:** 2024-03-21

**Authors:** Daniel Bravo, Ruth Quiroga-Mateus, Marcela López-Casallas, Shirley Torres, Ramiro Contreras, Andres Camilo Mendez Otero, Gustavo A. Araujo-Carrillo, Carlos E. González-Orozco

**Affiliations:** 1 Laboratory of Soil Microbiology and Calorimetry, Centro de Investigación Tibaitatá, Corporación Colombiana de Investigación Agropecuaria (AGROSAVIA), Km 14 Vía Bogotá-Mosquera, Cundinamarca, Colombia; 2 Centro de Investigación La Libertad, Corporación Colombiana de Investigación Agropecuaria (AGROSAVIA), Km 17 Vía Puerto López, Villavicencio, Meta Colombia; 3 Centro de Investigación Tibaitatá, Corporación Colombiana de Investigación Agropecuaria (AGROSAVIA), Km 14 Vía Bogotá-Mosquera, Cundinamarca, Colombia

**Keywords:** *Theobroma cacao* L., Cadmium distribution, Soil properties, Cocoa seeds, Artisan chocolate bars, Environmental monitoring

## Abstract

The district of Arauca is the second-largest producer of cacao in Colombia. However, despite its quality, it faces issues for export due to levels of cadmium (Cd) higher than the regulatory thresholds. A central question is how it may impact agricultural performance in the presence of Cd in cacao and chocolates. This study quantified Cd in cacao plantations from Arauca. Thus, 180 farms were assessed in the municipalities of Arauquita, Fortul, Saravena, and Tame. Five sample types (soil, irrigation channel sediment, soil litter, cacao seeds, and chocolates) were assessed for Cd. As a technological innovation, the new MXRF technology was used for Cd in chocolates. The sequence of Cd content was soil litter > chocolate > soils > cacao seeds > irrigation-channel sediment. A gradient north–south of Cd content in soil was observed, where highest content was found in farms near the Arauca River, and lower farther away. In irrigation channel sediment, Cd levels averaged 0.07 mg kg^−1^. The Cd content in cacao seeds was 0.78 mg kg^−1^ on average. Cd content in chocolates was above the threshold (1.10 mg kg^−1^ on average, including several cacao mass percentages). These artisanal chocolate bars produced by single farms were near the limit of Cd set by the European Union (up to 0.8 mg kg^−1^). Therefore, mixing beans from different farms could reduce their Cd content. The present study underscores the complexity of Cd distribution, emphasizing the importance of integrating soil, crop, and landscape features in managing and mitigating Cd levels in cacao.

**Supplementary Information:**

The online version contains supplementary material available at 10.1007/s10661-024-12539-9.

## Introduction

Colombia has 54 cacao-producing provinces with diverse agro-ecological characteristics, including climatic conditions, soil types, and cropping systems (González-Orozco & Pesca, 2022). Situated in eastern of Colombia, Arauca is one of the top cocoa producing regions of the country. The environmental conditions of Arauca are unique and favour cacao productivity. This has resulted in the production of fine and flavour cocoa beans, mainly derived from the ‘Criollo’ and ‘Trinitario’ cacao varieties found throughout the country (Rodriguez-Medina et al., 2019).

Arauca generates 11% of national production contributing 10 522 Mg of dry beans during last year (FEDECACAO, 2023). Nevertheless, cacao in Arauca has some challenges to increase its potential in competitivity. One of the challenges is the presence of cadmium (Cd) in the beans, and therefore in chocolates. This is an issue because the Commission of the European Union (EU) have established maximum permissible levels of Cd in chocolate and final products derived from cacao beans ranging from 0.1 to 0.8 mg kg^−1^ depending on the product (Commission, 2007; EU, E., 2014).

There are several definitions of heavy metals, as shown in a previous review (Duffus, 2002). However, in this study, we will refer to the concept of heavy metal as an element that has hazardous effects on some living systems, mainly in plants and humans. Cd is a non-essential element for almost all life systems, including humans, plants, and some microorganisms (Himeno et al., 2019; Satarug, 2019). Cd accumulation in soil and plants is due to (i.) geological sources (Chavez et al., 2015); and (ii.) anthropogenic influences (Bravo et al., 2021). In regard to soil, the dynamics of both soil pH and organic matter influence the Cd content in cacao beans (Kubier et al., 2019). The percentage of clay, and soil organic matter content (SOM) affects the mobility and concentration of Cd (Jovantheo et al., 2022). The importance of competition between other elements, nutritional status of the plant, age, and genetics, among others, also play a part (Bravo et al., 2018; Gil et al., 2022; Meter et al., 2019; Quiroga-Mateus et al., 2022).

At the national scale, preliminary studies described the causes of the presence of Cd in cocoa crops (Bravo & Braissant, 2022; Bravo et al., 2021). At the regional level, we still know little about Cd distribution and origin. Although there is some expert investigation related to cocoa and chocolate products elsewhere in Colombia (Bravo et al., 2022; Mounicou et al., 2002), these are just bright spots of knowledge regarding Cd origin and distribution. Another research gap is the absence of strategies to minimize the presence of Cd in the value chain once it has been identified. This is important to resolve to ensure that any results are reliable and representative, especially in relation to trade requirements.

In Arauca, since 2016, a few studies have been carried out to determine Cd concentration in some farms (Aristizabal-Aristizabal, 2021; Bravo, 2022; Bravo et al., 2018, 2021; Charrupi-Riascos, 2018). In 2017, a study by the University of La Salle, was carried out on Cd and its availability to cacao in the Arauca and Nariño regions (Aristizabal-Aristizabal, 2021). The studies found that, in Arauca, the presence of Cd in soils is near (above than the recommended level) to the recommended normal rates for agricultural soils of 1 mg kg^−1^, as previously found (Bravo & Benavides-Erazo, 2020), with Cd concentrations of 0.98, 1.0, 1.38, 1.39, and 1.4 mg kg^−1^ in the municipalities of Arauca, Arauquita, Fortul, Saravena, and Tame, respectively. A set of previous studies have been performed by AGROSAVIA, the Colombian Research Centre for Agriculture. For instance, a study was performed in the northeast of Colombia (Bravo et al., 2018) focused on understanding the role of cadmium-tolerant bacteria (CdtB) in cacao fields. Another study developed a diagnostic method based on soil geophysical properties such as resistivity and soil parameters to assess Cd distribution in subsurface soil of cacao farms (Bravo & Benavides-Erazo, 2020). A recent study shows the uses of isothermal microcalorimetry (IMC), to measure metabolic activity of CdtB in soils through heat-flow thermodynamically analysed (Bravo, 2022). Furthermore, FEDECACAO, the National Federation for Cacao Farmers, performed a preliminary soil survey in 446 cacao farms of the municipalities of Arauca, Arauquita, Saravena, Fortul, and Tame (FEDECACAO, 2023).

Nonetheless, to date, there is no field study integrating both Cd uptake in cacao and aspects such as geology and geochemistry in Arauca. Besides, no study has focused on investigating the effects of such variables on the final products (chocolates) as we propose. Therefore, the aim of this study was to quantify the cadmium content in several matrices of cacao farm-systems in Arauca and evaluate the relationship with soil properties, such as the pH, SOM, the content of major elements, as well as the relation with geological and soil cartographic units, to understand the content of cadmium in cacao beans and artisanal chocolates. Our study shows (i.) how the distribution of Cd could be affected by geological or soil conditions across Arauca, and (ii.) that the levels result in chocolate with cadmium near to the specified thresholds for trade. Moreover, our approach helps to enhance our current knowledge on the potential effects of geological and soil conditions as a driver of Cd accumulation in cacao crops. At the end, this paper compares the Cd content in both cocoa beans (raw material) and chocolate bars (final product) made in the region, in terms of Cd distribution.

## Materials and methods

### Study area

**Fig. 1:**
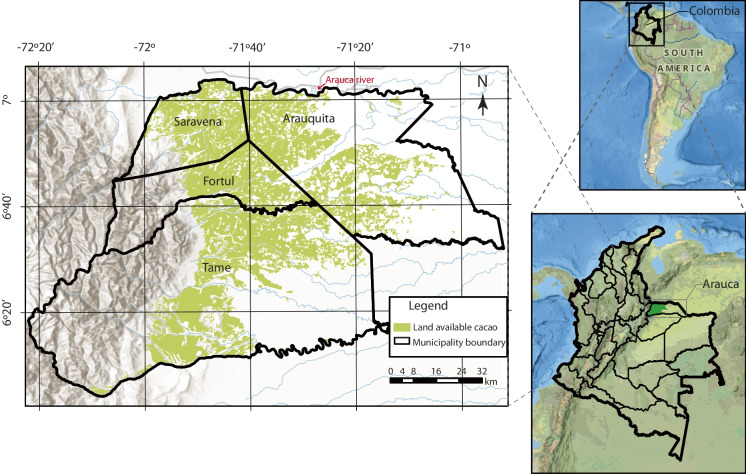
Study area of cadmium in cacao crops. The map includes the land suitability layer for cacao in Colombia, adapted from the Rural Agricultural Planning Unit (UPRA, 2018). This study included farms assessed from four municipalities: Saravena, Fortul, Tame, and Arauquita

The study area is in eastern side of Colombia, in the region of Orinoquía (Eastern Plains), within the district of Arauca. The study includes cacao farms from four municipalities: Saravena, Arauquita, Fortul, and Tame (Fig. 1). Quaternary alluvial and floodplain deposits are the main chronostratigraphic units. Moreover, there are four main landscapes: mountains, foothills, valleys, and floodplains, where the latter is associated with the Arauca River in the north of the region (IGAC I., 2015).

### *Field sampling*

For a diagnosis of Cd content in Araucan cacao crops, samples of soil, soil litter, and irrigation channel sediment were collected. A total of 180 cacao farms were sampled for soils and litter, 20 and 14 sampled farms for irrigation channel sediment and cacao seeds, respectively. All the samples were allocated among the four municipalities. The 180 farms were selected based on Cd soil hotspots previously determined (Bravo et al., 2021) and using an agroclimatic approach (González-Orozco et al., 2023). The sampling points were delimited physically due to the evaluation area by cacao coverture of both genetic material diversity and contrasting soil Cd content, based in a previous study (Bravo, 2022).

Since it was an agronomic study and not a soil quality study, the method for sampling consisted of collecting a composite sample of 1 kg from the first 30 cm of soil depth, 70 cm from the trunk of the tree. The samples were taken in zig-zag from ten cocoa trees delimited in 1 ha. The sampling of litter samples was carried out in a zig-zag pattern, collecting five subsamples per hectare (ha). One kilogram of composite soil litter was collected. To this, a minimum of 200 g per subsample was required to ensure the enough mass for Cd quantification.

The irrigation channel sediment samples were collected for 20 farms. The points selected for sampling were defined by the location of the irrigation channels. One sample was obtained near the water inlet, and another sample was collected near the water outlet, in each of the farms.

Moreover, 14 farms were selected to perform the quantification of Cd in fresh cacao seeds. This selection was based on the availability of ripe fruit when the sampling was carried out. Four cacao pods per tree were selected randomly from a hectare at each farm. The cacao varieties assessed in this study are shown in Table 1.

**Table 1:** Cacao varieties found in the selected 180 cacao farms to this study in four municipalities of Arauca. The samples were performed in dry season

No. farms	Municipality	Cacao variety	Range of age [years]	Altitude average[meters]	Intercropping trees
60	Arauquita	FSA 13, FEAR 5, FTA 2, FTA 4, FSV 41, FEC 2, CAU 39, FLE 2, ICS 1, SCC, HIBRIDOS	3–20	144–160	*Persea americana, Erythrina poeppigiana, Samanea saman, Cedrela odorata, Cordia alliodora, Citrus sinensis, Gmelina arborea, Acacia Mangium, Annona muricata, Gliricidia sepium, Quararibea cordata, Anacardium excelsum, Tabebuia rosea, Cariniana pyriformis* *Mangifera indica, Eugenia stipitata, Tectona grandis, Ficus dendrocida, Handroanthus chrysanthus, Pourouma cecropiifolia, Leucaena leucocephala, Swietenia macrophylla, Pachira quinata*
46	Saravena	FSA 13, FEAR 5, FTA 2, FSV 41, FEC 2, FTA 4, CCN 51	2–20	187–201	*Erythrina poeppigiana, Samanea saman, Cedrela odorata, Cordia alliodora, Gliricidia sepium*
34	Fortul	FSA 13, FEAR 5, FTA 2, FTA 4, FEC 2, FLE 2, FSV 41, CCN 51	3–20	193–201	*Persea americana, Erythrina poeppigiana, Samanea saman, Cedrela odorata, Cordia alliodora, Gliricidia sepium*
40	Tame	FSA 13, FEAR 5, FTA 2, FTA 4, FEC 2, FSV 41, CCN 51	3–15	174–272	*Persea americana, Erythrina poeppigiana, Citrus sinensis, Cedrela odorata, Matisia cordata, Cordia alliodora, Samanea saman*, *Acacia mangium*

Moreover, six samples of artisanal chocolate manufactured locally by cooperatives from single farms were also analysed for Cd. The chocolate was made with beans from the farms being studied. The artisan chocolate was made mixing three units from the same production batch. All the chocolate samples were prepared in the same farms.

### Cartographic distribution

Two sources of information were used to identify the spatial distribution of chosen environmental variables. The sources of information refer to two available studies developed by Colombian agencies: IGAC (Agustin Codazzi Geographic Institute) and SGC (Colombia Geological Service). The first was the general soil survey for Arauca region at a scale of 1:100,000 developed by the Instituto Geográfico Agustín Codazzi (IGAC, 2017). This study includes the cartographic soil units (CSU) and their major features. The second was the geological map at a scale of 1:100,000 carried out by the Servicio Geológico Colombiano (SGC, 2015). Both studies have a series of delineated polygons based on qualitative characteristics including parent material and landscape. Geoprocessing of polygons was performed using ArcGIS Pro software version 2.9.0 (ESRI, 2021).

### Physicochemical parameters in the cacao system

The quantification of physicochemical parameters in both soils and plant-tissue samples was carried out in the Analytical Chemistry Laboratory of Agrosavia, located in Research Centre Tibaitatá, in Mosquera, Colombia. To the soil samples, the pH was analysed using a potentiometric method, according to a previous method (Peech, 1965). The acidity (Al^+^H) was determined by the volumetric method (Coleman & Thomas, 1967) using a T90 Automatic Titrator (Mettler Toledo, Columbus, OH, USA). The aluminium (Al) content was determined by atomic absorption spectrometry—AAS (Agilent 280FS AA, California, USA), dissolving the soil in a KCl solution according to the reported method (Pratt & Bair, 1961). Electrical conductivity (EC) was determined using the method proposed previously (Richards, 1954). The organic carbon content of the soil (OC) and SOM was determined by oxidation with potassium dichromate and sulphuric acid using the modified method (Walkley & Black, 1934) and quantified by UV–visible spectrophotometry (Perkin Elmer Lambda 25 spectrometer, Waltham, MA, USA). Exchangeable cations Ca^2+^, K^+^, Mg^2+^ were extracted using a solution of CH_3_COONH_4_ 1 M at pH 7.0 by the method proposed in a previous study (Shuman & Duncan, 1990). The extracted elements were quantified using AAS. Minor elements Fe, Cu, Zn, and Mn were quantified by the standardized method for soils from the Colombian eastern plains, using DTPA pH 7.3 extractant solution (Lindsay & Norvell, 1978) and determined by AAS. The phosphorus (P) content was determined by reduction with ascorbic acid using the Bray II method (Bray & Kurtz, 1945).

### Cadmium determination

To all cadmium quantifications of the sample types below described, the measurements were performed using biological replicates (*n* = 3). Moreover, to each composite sample, it was performed statistical replicates of lectures (*n* = 2) validating both, spectrometric (ICP-MS) (Mounicou et al., 2002) and Monochromatic Energy-Dispersive X-Ray Fluorescence (MXRF®) (Chen et al., 2008) technologies.

#### In soils

Cadmium was determined using an inductive-coupling plasma spectrometry coupled with mass spectrometry (ICP-MS, Agilent 2000 Series Agilent Technologies, USA). Before carrying out the digestion, mineralization, and quantification process, all the samples went through a drying, grinding, and sieving processes. The drying was carried out in a drying oven with a temperature control not higher than 40 °C, to preserve all the chemical characteristics. The electrical grinder used for powdering the samples was a Kitchenaid BCG111 made of polycarbonate (Whirlpool, London, UK). The grinding process was carried out in a hammer mill with a 2-mm mesh opening, to obtain a homogeneous sample; then, the sample was sieved with a 0.5 mm of opening sieve. The mineralization process was carried out by a double acid wet route, nitric-hydrochloric acid digestion according to a previous study (Bravo et al., 2018; Rodríguez Giraldo et al., 2022). Pseudototal Cd quantification was performed by direct lecture in ICP-MS, matching the acceptance criteria, calibration curve (*r* ≥ 0.995) and recovery percentages between 85 and 115% (Rodríguez Giraldo et al., 2022). Spiked solutions were measured during the process with recovery percentages between 85 and 115%, using the certified cadmium reference material traceable SRM NIST® SRM® Loam soil (trace elements), ERM—CC141 (Merck Germany) and the reference material—Clay WEPAL-ISE-961 (International Soil-Analytical Exchange (ISE)/The Wageningen Evaluating Programme for Analytical Laboratories—WEPAL).

#### In irrigation channel sediment

The available Cd content in irrigation channel sediment was determined by ICP-MS. Particularly in irrigation channel sediment, due to the high humidity of the samples, a longer-time drying in an oven than soil samples were necessary to limit microbial activity (including Cd immobilization one) and avoid sample alteration. Due to the higher sand contents, the samples were dried at a temperature not higher than 40 °C for 24 h. After the pre-treatment, all the samples were mineralized by double acid digestion, applying the same methodology for the soil sample (Rodríguez Giraldo et al., 2022; USEPA 1996). Cadmium concentrations in irrigation channel sediment may vary from 0 to 16 mg kg^−1^ (Chavez et al., 2015), where upper than 0.5 mg kg^−1^ of available Cd is considered polluted. As quality control of the quantification process, the reference materials used were the same as those for soil analysis.

#### In soil litter

Cd content of soil litter was measured according to a previous method reported (Gil et al., 2022). The samples were first washed with a 0.1 M HCl solution and rinsed with deionized water for 30 s. Then, the samples were dried in an oven at 68 °C for 48 h, and then the dry material was homogenized in a mill. Later, 0.50 g of sample was digested with a solution of HNO_3_-HCl_3_O_4_ (5:2 v/v) using a microwave digester (Milestone ultraWAVE, Sorisole, Bergamo, Italy). The Cd content was also determined by ICP-MS. To guarantee the veracity and quality of the data, efficiency was determined in recovery percentages between 85 and 115% of the standard reference material of trace elements in spinach leaves (NIST® SRM® 1570a).

#### In cacao seeds

Cd quantification in cacao seeds was measured by ICP-MS under the method above-mentioned (Rodríguez Giraldo et al., 2022). This protocol was validated in previous O-ring tests for cacao beans. The limits of Cd detection (LOD) and Cd quantification (LOQ) were 0.005 mg kg ^1^ and 0.043 mg kg ^1^, respectively. The Cd recovery percentage was 92%, using a certified bakery chocolate reference material (chocolate NIST® SRM® 2384).

#### In chocolate bars

Cd was quantified using the ICP-MS equipment. In a first step, the whole chocolate bar sample was ground. Later, a portion of the homogeneous sample was taken for Cd analysis from a quartering method (Byrnes, 2008). The samples were digested according to a previous methodology (Bravo et al., 2022; Engbersen et al., 2019). Moreover, a new method was used in this sample type. Cd was quantified using the monochromatic X-ray fluorescence (Chen et al., 2008) also known as MXRF technology (E-max, Z-Spec, East Greenbush, NY, USA). To this, the chocolate samples were cooled at 4 °C for 24 h. Once the powder was collected, 0.5 g of powder was sealed into a single polycarbonate tube. The tube was inserted in the E-max equipment horizontally, for reading. The read time was set to 200 s, and the readings were made in duplicate. In both cases (ICP-MS and MXRF), the lectures were compared to certified reference material (chocolate NIST® SRM® 2384).

### Statistical analysis

A descriptive and exploratory analysis was performed with all Cd sample types (Cd content in soil, soil litter, cacao beans, and soil irrigation channel sediment). The analysis included the calculation of central tendency measurement and data dispersion. Normal distribution was checked and corrected if needed. Furthermore, a one-way analysis of variance (ANOVA) and Tukey comparison were performed with pseudo-total concentration of Cd in soil (or its transformation) and the categorical environmental variables (soil units, and geological units). The compute parameters were performed with *α* = 0.05.

A Pearson lineal correlation (*r*) was made between the raw data of all physico-chemical parameters vs. Cd in soils. The analysis included a comparison of greater correlation between the selected parameters and Cd to understand the relationship of the distribution of the parameters with the presence of Cd mainly in soil. To analyse the relationship between Cd in soils and soil parameters, a correlogram plot was designed that shows only linear correlations with *p value* < 0.05. Moreover, a log-10 scale plot was performed to fit Cd from four out of five sample-types measured in this study to observe the fluctuation of the heavy metal across several steps of the cacao value chain. Cd content measured in chocolate was described separately taking into account the limit imposed by the EU (European, 2014).

All data processing and testing were carried out in R software version 4.1.2 (Team RC, 2021). The statistical plots were performed using QtiPlot vs. 2022 5.12.8. The sketches were edited using the vector graphics editor and design program package of Adobe Illustrator (Ai) vr. 27.2, 2023. The base-maps used were obtained by the service layers from ESRI, FAO, NOAA, and USGS, and all the maps were reported according to these layers.

## Results

### Geological features

Specific soil properties were obtained from sampling points. The soil characterization expressed in Table 2 summarizes the main soil chemical properties in the study area. According to chemistry data, the Arauca soils had the following characteristics regarding the guidelines developed by ICA (1992) and IGAC (2014): moderately acidic soils ranging 5.5 to 6.0, low SOM content (< 2.0%), a normal EC for agriculture in Colombia (< 2 dS m^−1^), medium P content, ranging 15 to 40 mg kg^−1^, low ECEC (< 10 meq 100 g^−1^), medium Ca content, ranging 3 to 6 meq 100 g^−1^, low Mg content (< 1.5 meq 100 g^−1^), low K content (< 0.20 meq 100 g^−1^), and low Na content (< 1 meq 100 g^−1^). Regarding the minor elements the soils showed a good Zn content, ranging 3 to 6 mg kg^−1^, low Cu content (< 1.5 mg kg^−1^), optimum Mn content, ranging 15 to 30 mg kg^−1^, low B content (< 0.20 mg kg^−1^), and an excess of Fe (> 30 mg kg^−1^).

**Table 2:** Statistical description of main soil chemical properties in soil samples distributed in the study area

Soil property	Unit	n	Mean	Min	Max	SD	CV [%]	Skewness	Kurtosis
B	mg kg^−1^	180	0.18	0.01	1.05	0.14	77.54	2.32	9.66
Ca	meq 100 g^−1^	180	3.57	0.6	10.11	1.49	41.9	0.65	0.97
Cu	mg kg^−1^	180	1.09	0.2	3.91	0.68	62.13	0.72	0.5
EC	dS m^−1^	180	0.17	0.08	1.46	0.12	71.41	7.01	65.26
ECEC	meq 100 g^−1^	180	5.7	2.22	11.72	1.78	31.23	0.49	− 0.09
Fe	mg kg^−1^	180	87.01	29.06	253.46	34.63	39.79	0.87	2.18
K	meq 100 g^−1^	180	0.14	0.06	0.5	0.09	64.51	1.72	3.16
Mg	meq 100 g^−1^	180	1.46	0.22	3.11	0.68	46.56	0.36	− 0.58
Mn	mg kg^−1^	180	20.15	5.47	46.84	7.68	38.15	0.69	0.76
Na	meq 100 g^−1^	180	0.062	0.06	0.26	0.02	31.33	8.34	71.75
P	mg kg^−1^	180	16.16	1.86	104.6	18.87	116.76	2.36	5.91
pH	unit	180	5.55	4.62	7.31	0.42	7.49	0.72	1.7
SOM	%	180	1.38	0.5	2.5	0.45	32.33	0.34	− 0.5
Zn	mg kg^−1^	180	5.22	1.28	29.66	2.89	55.3	4.08	28.72

**Fig. 2:**
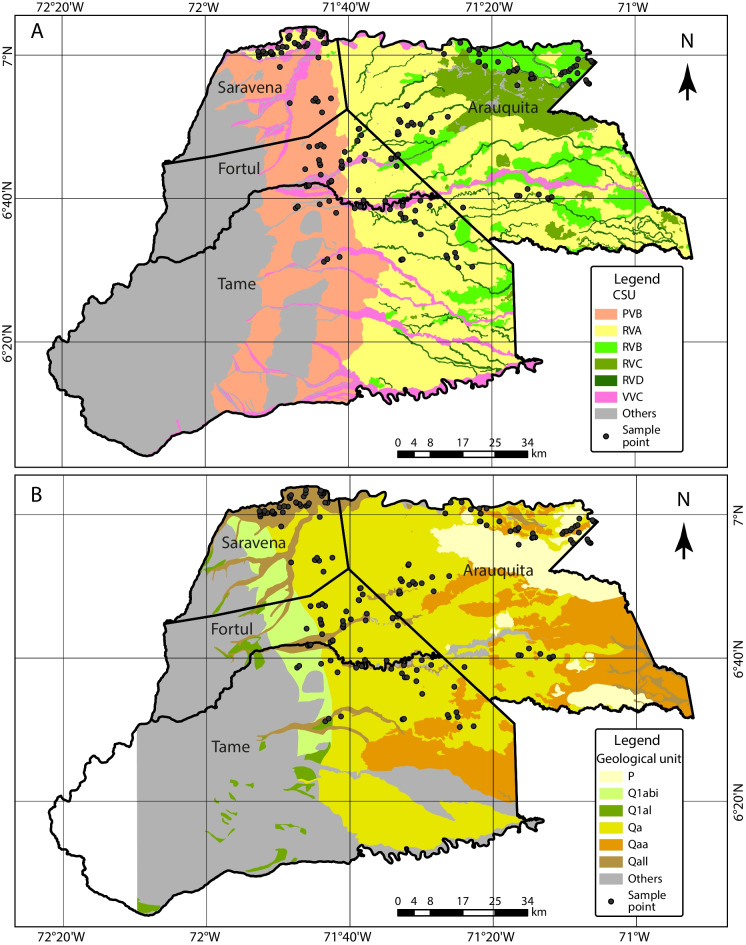
Cartographic soil units (CSU) (**A**) and geological units (**B**) in Arauca. PVB = alluvial cones in foothill; RVA = terraces in floodplain; RVB = overflow plains in floodplain; RVC = hollows in floodplain; RVD = valleys in floodplain; VVC = planes in alluvial valley. P = borders of wetlands; Q1abi = lower fan deposits; Q1al = ancient alluvial deposits; Qa = alluvial deposits on extensive plains; Qaa = alluvial floodplain deposits associated with organic material; Qall = floodplain deposits. Others = others CSU or geological unit

Figure 2 shows the overlapping of both soil and geological cartographic units with the location of the farms. The 180 sampling points (farms) were distributed in six CSU (Fig. 2A). One unit in the landscape of foothill (PVB [alluvial cone] ~ 30 samples), four units in the landscape of floodplain (RVA [terrace] ~ 94, RVB [overflow plain] ~ 22, RVC [hollow] ~ 8, RVD [valleys] ~ 12 samples), and one unit in the landscape of alluvial valley (VVC [plane] ~ 14 samples). Around 75.6% of the samples were found in the floodplain landscape, the flat area bordering mainly the Arauca River. Moreover, six geological units composed the sampling points (Fig. 2B): lower fan deposits (Q1abi ~ 4 samples), ancient alluvial deposits (Q1al ~ 1 sample), alluvial deposits on extensive plains (Qa ~ 105 samples), alluvial floodplain deposits associated with organic material (Qaa ~ 5 samples), floodplain deposits (Qall ~ 53 samples), and borders of wetlands (seasonal systems) (P ~ 12 samples); 58.3% of the samples were identified as Qa, a unit distributed in a major part of the study area (mainly in the central and eastern regions) and formed on thick layers of fine to very fine quartz sands, angular, with light gray muds and clays (SGC, 2015).

### Cadmium content in cacao farms of Arauca

The descriptive and exploratory analysis was summarized in Table 3 and 4. The largest amount of Cd data was in soils and soil litter (*n* = 180), while the least was in chocolates (*n* = 6). The coefficient variation (CV) was up to 77.3% (Cd pseudo total in irrigation channel sediment of the entry points). Except for Cd in seeds (*n* = 14), all sample types had an asymmetrical (positive) distribution skewed to the right (skewness > 0) and the majority suggesting a leptokurtic distribution (kurtosis > 0). As follows, each of the sample type is described.

**Table 3:** Statistical description of cadmium concentration in several sample types in the studied area

Sample type [mg kg^−1^]	n	Mean	Min	Max	SD	CV [%]	Skewness	Kurtosis
Cd pseudo-total in soils	180	0.48	0.04	2.31	0.37	77.2	1.55	3.07
Cd in soil litter	180	9.86	0.00	36.02	6.53	66.2	1.02	1.06
Cd in seeds	14	0.78	0.32	1.19	0.24	45.2	− 0.18	− 1.04
Cd pseudo-total in irrigation channel sediment entries	20	0.57	0.10	2.16	0.44	77.3	2.28	5.90
Cd available in irrigation channel sediment entries	20	0.06	0.01	0.17	0.05	70.9	0.72	− 0.62
Cd pseudo-total in irrigation channel sediment outputs	20	0.56	0.06	1.55	0.36	64.5	1.01	0.60
Cd available in irrigation channel sediment outputs	20	0.07	0.01	0.25	0.07	87.4	1.30	0.96

**Table 4:** Cd content in artisanal chocolate bars found in Arauca

ID	Cacao mass [%]	Cd E-max	SD Cd E-max	Cd ICP-OES	SD Cd ICP-OES	Cd average
		mg kg−1				
2023_C2	100	1.098	0.025	0.886	0.072	**0.992**
2023_C3	68	1.109	0.014	0.928	0.076	**1.019**
2023_C4	100	0.776	0.026	0.396	0.032	**0.586**
2023_C5	70	1.452	0.045	1.192	0.097	**1.322**
2023_C6	90	0.849	0.035	0.581	0.047	**0.715**
2023_C7	70	2.195	0.045	1.831	0.090	**2.013**

### In soils

**Fig. 3:**
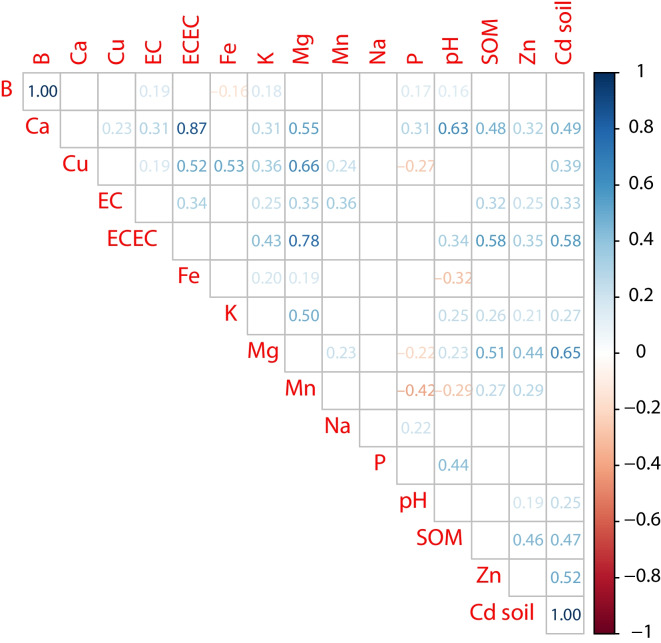
Correlogram between main soil elements and properties and pseudo-total Cd concentration in soils in the study area. Figure depicts the correlation coefficients with *p value* < 0.05

The correlogram shown in Fig. 3 describes the relationship between the pseudo-total Cd concentration in soils and the main soil elements and properties in the study area. The highest correlations (correlation coefficient (*r*) > 0.4 and *p value* < 0.05) were: Mg (0.65), ECEC (0.58), Zn (0.52), Ca (0.49), and SOM (0.47). An approximation from the municipalities showed that in Arauquita, the highest correlation was found with SOM (0.70), Mg (0.67), and Zn (0.52). In Fortul, the highest correlation was found with Ca (0.59), Mg (0.55), and Zn (0.51). In Saravena, a higher correlation to Cd was found with ECEC (0.62), Mg (0.53), and Ca (0.50). Besides, in Tame it was found a high correlation between soil Cd with Zn (0.80), Ca (0.73), and ECEC (0.62).

**Fig. 4:**
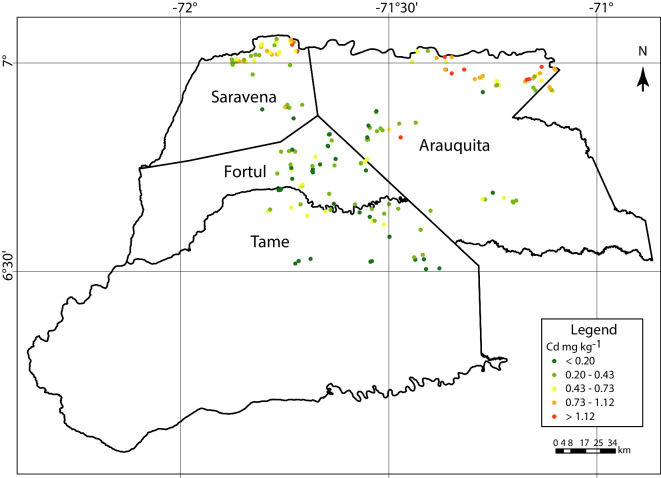
Geographical distribution of pseudo-total Cd in soils from the 180 assessed farms in Arauca

The geographic distribution of soil Cd pseudo-total content is shown in Fig. 4. The data were classified in five categories generated by Natural Breaks (Jenks) ranged from < 0.20 to > 1.12 mg kg^−1^. The highest concentrations were found in the north, and the lowest and medium concentrations were found in the centre and south of the region, respectively. Looking at the distribution of Cd in function of the municipalities the higher Cd in soils found was in Arauquita (0.65 mg kg^−1^ on average).

**Fig. 5:**
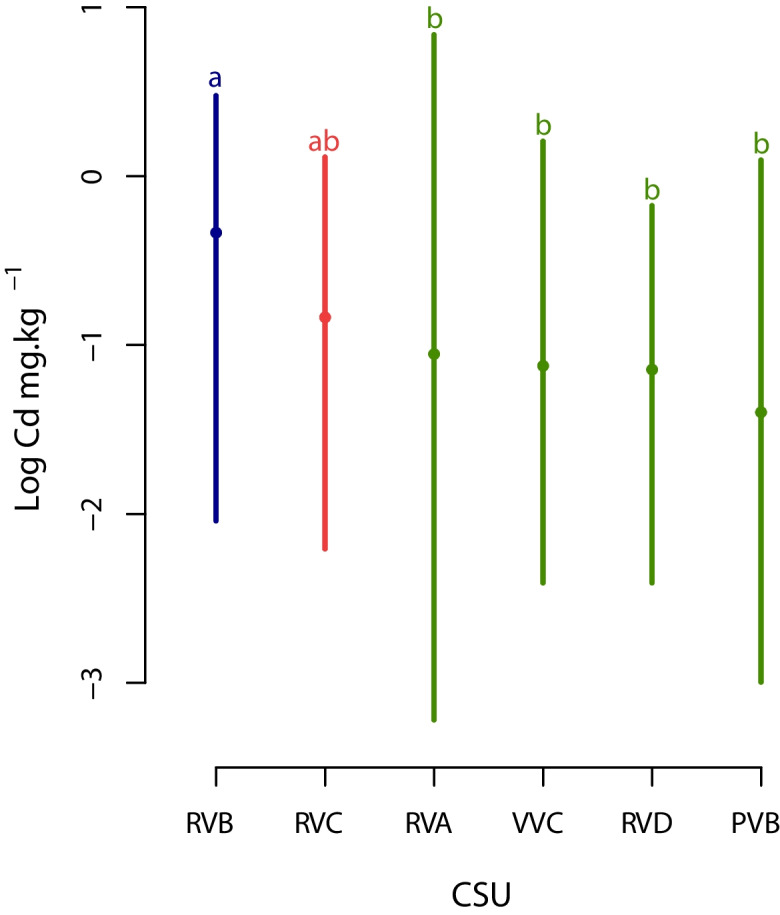
Multiple comparisons in Cd pseudo total in the soil and cartographic soil units by component. Different bar colours mean statistical differences of Tukey test assessed. The dot on each bar represents the average concentration per cartographic soil unit. The Y axis is shown in log values. A statistically significant difference was observed (F(1) = 5.45, *p* < 0.05

Since the pseudo-total Cd concentration in soils did not have a normal distribution, with a skewed right distribution (Table 3), it was transformed using a logarithmic function. We found a statistically significant difference in average log Cd concentration in soil units (F(1) = 5.45, *p* < 0.05). However, in terms of geological units no differences were found (F(1) = 2.26, *p* > 0.05). Regarding multiple comparisons of groups by means of Tukey the results indicated that CSU were grouped in three groups, expressed in the Fig. 5 in logarithmic scale: the ‘a’ class with RVB unit (0.84 mg kg^−1^), the ‘ab’ class with RVC unit (0.56 mg kg^−1^), and the ‘b’ class with RVA, VVC, RVD, and PVB units (0.42 mg kg^−1^). Looking at the CSU, the RVB had the highest concentration (Fig. 2A). On the contrary, geological units were not analysed because not statistically significant differences were found (see Fig. 2B). The distribution of pseudo-total Cd concentration in soils regarding CSUs is shown in supplementary Figure S1.

### In irrigation channel sediment

The soil irrigation channel sediment showed an average Cd available content lower than 0.06 mg kg^−1^. While in 95% of the samples assessed there were no detected differences between the Cd of irrigation channel sediment from the entry and exit points of the farms (± 0.07, in standard deviation of the replicates at each point) it was found two out-layers related to high Cd content in both soils and cacao beans. Table 3 shows both the entry and out sampling points of soil irrigation channel sediment. The entry points had an average content of 0.05 mg kg^−1^ of Cd. The exit points had an average of 0.06 mg kg^−1^ of Cd. The geographical distribution of available Cd in irrigation channel sediment is shown in supplementary Figure S2.

### In soil litter

The values found in soil litter exhibited variable ranges. The average value of Cd content was 10.03 mg kg^−1^. The maximum value was 36.02 mg kg^−1^ corresponding to a percentage of dry matter of 84.53%, and a value of 0.54 mg kg^−1^ was the lowest content. The distribution of Cd in soil litter regarding municipalities is shown in supplementary Figure S3. The correlation coefficient between Cd in soils and Cd in soil litter showed a positive correlation between these variables (*r* = 0.55, Pearson coefficient).

### In cacao seeds

According to the 14 cacao seeds samples, the highest Cd content found was 1.19 mg kg^−1^, while the minimum value was 0.32 mg kg^−1^ and a 0.78 value was found on average. Interestingly, there were no significative differences between Cd content between the municipalities, indicating low variation in the region. That said, it was seen in the geographical distribution, a slight variation of Cd in the gradient from north (higher) to south (lower) of the Arauca district. It is noteworthy, that the sampling of fresh cacao beans was done in cacao farms belonging to all the municipalities here assessed.

Five out of 14 were sampled in Arauquita, showing a 0.9 mg kg^−1^ of Cd content in seeds, on average. Four samples of them of Saravena showed a Cd content of 0.81 mg kg^−1^ on average. Three and two out of 14 samples were coming from Fortul and Tame municipalities, with 0.69 and 0.68 mg kg^−1^ of Cd contents, respectively. The pods assessed belong to both international and regional cacao varieties. The regional varieties are known as the ‘Araucan model’ and comprise the materials of Arauquita 5, Saravena 13, and Tame 2. The common universal material was CCN 51 (see Table 1).

**Fig. 6:**
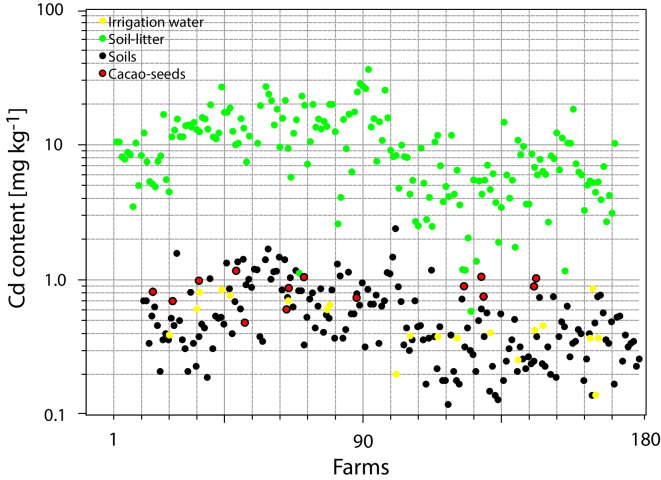
Cd content in several sample types of the cacao/chocolate chain value in Arauca. The sketch shown the flux of Cd from soil samples to cacao powder within the assessed farms. The ‘y’ axis is shown in log10

The Cd content on fresh seeds was included in the comparison of four Cd sample types described in Fig. 6. In such figure, it is exhibited the relation between Cd content in the four out of five sample types here studied. Even though, measuring Cd in cacao beans is a challenge, due to the heterogeneity of the composite samples of the farms assessed in this study, the average Cd content found was below to the limit imposed by the European regulation up to 0.8 mg ± 0.2 kg^−1^. Interestingly, the lower Cd content detected in fresh seeds was 0.32 mg ± 0.2 kg^−1^.

### In chocolate bars

The Cd values for six chocolate bars are shown in Table 4. The table includes the data of quantification in both ICP-OES spectrometry and MXRF fluorescence technologies. An average is shown because of all measurements with both available technologies in this study. The range of Cd in chocolate was 0.58–2.01 mg kg ^1^. Although four out of the six samples assessed showed Cd values above the European limit (0.8 mg kg^−1^ for samples with 50 ≥ 70% cocoa mass), three of them were near (above or below) to the limit of 0.8 mg kg^−1^ with a maximum variance of ± 0.2.

## Discussion

Before this study, there was not enough information or data to support any discussion for this area in a local context. Therefore, this study shows the Cd variations at the district scale, providing baseline information about how to develop strategies of Cd mitigation in the cacao crop of Arauca. This is the first major characterization of the Cd content in the whole local chain, highlighting the average, the limit, and the expected values, in terms of final consumer impact. Thus, understanding how geology, soil conditions, Cd in sediments landscape, and flooding have an effect on the Cd content in cacao beans, and consequently, in chocolates, is the first step in strategies of mitigation for the cacao value chain.

### Role of soil and geology on Cd in soil distribution

The soil characteristics obtained from the soil samples were like those evaluated by León-Moreno et al. (2019) using 59 samples in Arauca. For example, K had the same average (0.14 meq 100 g^−1^), while pH had a difference of 0.30, SOM 0.10%, P 0.22 mg kg^−1^, Ca 0.24 meq 100 g^−1^, Mg 0.24 meq 100 g^−1^, and ECEC a difference of 0.38 meq 100 g^−1^. Based on the minimum suggested levels by León-Moreno et al. (2019) for productive cacao farms, the soil characteristics in Arauca showed deficiencies, especially the soil variables SOM, Ca, K, and ECEC.

Regarding the correlation between pseudo-total Cd in soils and soil characteristics, the highest correlations were found with Mg, ECEC, Zn, Ca, and SOM. Although some authors indicate the common relationship between soil pH and Cd in soils (Argüello et al., 2019; Bravo et al., 2014; Scaccabarozzi et al., 2020), surprisingly, a weak correlation (*r* = 0.25 and *p value* < 0.05) was shown in this study (see Table 2 and supplementary Fig. 4). However, this result is similar to the findings obtained by Carrillo et al. (2023) in cacao productive regions in Costa Rica. A positive linear correlation with Mg, Ca, and ECEC (sum of previous cations plus K^+^ and Na^+^) has been reported in other regions in Colombia (Bravo et al., 2014, 2021), and in other cacao growing soils in the South American region (Chavez et al., 2015; Thomas et al., 2023). According to Li et al. (2022), Ca is an essential macronutrient and divalent cation, and they suggested that Cd could enter plant cells through the Ca^2+^ ions channels. The same authors indicate that Mg competes with heavy metal ions during the root uptake process and affects the accumulation and transport of Cd in crops such as rice. The ‘good Zn content term’ was considered based on the physicochemical tests performed in the laboratory of Analytical Chemistry at Agrosavia, in comparison with the standards released by the national reference entity, the Colombian Agricultural Institute (Rojas et al., 1992). It is worth mentioning that this national standard aligns with the international context of Zn for cacao (Wessel, 1980). The good correlation with Zn could be explained because they share very similar chemical properties (Jiménez Tobón, 2015). Although Cd is similar to Zn and the former always occurs in combination with the latter, Cd forms more complex compounds (Vasiliu & Dixon, 2018). The SOM content evidenced a positive correlation. Smolders and Mertens (2013) indicated that SOM is generally the second statistical soil parameter associated with Cd concentrations in solution and is part of the three main Cd adsorbents in soils. However, Li et al. (2024) highlighted that the effect of SOM on soil Cd availability may be significant or insignificant, and this contradiction may be attributed to the differences in SOM content (such as humic acid) and their composition in our findings.

The statistical results showed that from a geological point of view the Cd soil concentration is not related to geological layers described by SGC (2015) in the study area. However, the CSUs had significant differences from each other (Fig. 5), especially the RVB unit, which had the highest concentration, with a 0.84 mg kg^−1^ on average, followed by the RVC unit with 0.56 mg kg^−1^. The RVB unit is characterized by the overflow effect generated on the floodplain by the fluvial dynamics. Glacio-fluvial sediments from the Andes Mountain are deposited in this unit in fine sediments (clay and silt), mainly kaolinitic clays (more than 50%) of low activity (IGAC, 2017). The RVB unit has a singular geographic position in the study area, mainly in the northeast in an area called ‘Las Islas’, in the municipality of Arauquita, surrounding the mainstream and branches of the Arauca River, a site influenced by deposits from the Andes Mountain (mainly by the mountain complex called Montaña Santandereana). This finding is similar to the study by Gramlich et al. (2018), who indicated that high soil Cd concentrations in cacao farms were associated with alluvial soils from sedimentary materials (such as presented on the RVB unit), which can be explained by the higher Cd concentrations usually found in sedimentary rocks compared to igneous rocks.

### Cd rich sediments for farming

While this study did not sample rock types, nor water samples from river surface water, it was possible to measure Cd in the irrigation channel sediment on farms, close to Arauca River. In the irrigation channel sediments, higher concentrations of available Cd were found compared to other areas in Colombia, such as the irrigation districts in Magdalena River (Martínez-Mera et al., 2019) or in the west side of the Magdalena basin (Gil et al., 2022) with 0.03 and 0.04 mg L^−1^, respectively of Cd. In another study, focussing on heavy metal presence in agricultural sediments located in Colombia, for instance, in the Tunjuelo and Chicu Rivers the Cd content in sediments was between 0.5 and 13 mg kg^−1^ Cd on average, respectively, and in their surface water between 0.04 and 0.03 mg L^−1^ of available Cd, on average, respectively (Arias Espana et al., 2018). Thus, our findings of 0.05 and 0.06 mg L^−1^ suggest a sedimentation process of Cd may be occurring actively within the cacao crop soils (see also supplementary Figure S2).

### Role of landscape in Cd distribution

A latitudinal gradient in climate exists in Arauca (González-Orozco et al., 2023). Therefore, other variables that may have an influence on the Cd concentration in soils are climatic variables (Scaccabarozzi et al., 2020; Thomas et al., 2023; Tudoreanu & Phillips, 2004; Yi et al., 2020), which were not specifically evaluated in this study either and should be considered in further studies. Said that, the northern zone of Arauca, such as Saravena and Arauquita, presents distinctive climatic regions compared to the rest of the district, with rainier conditions in floodplain areas located near the foothills of the mountains. Conversely, as one moves southwards towards the interior areas of the territory in the municipalities of Tame and the southern part of Arauquita, the climate conditions change, characterized by being hotter and drier. Additionally, these areas are more hilly and are not necessarily prone to flooding. Our results of Cd presence in the soil indicated a north–south pattern, which could be influenced by these differential climate conditions observed changing from north to south. (ATSDR, 2012; Bravo et al., 2021; Kumar et al., 2019; Sabet Aghlidi et al., 2020).

The highest Cd concentration in sediments found on the coast, mainly in an area called ‘Las Islas’ (Figure S2), formed possibly in the early ‘Cretaceous Syn-Rift’ sedimentation in the Arauca Graben in the Llanos Basin, across the Arauca River, may disperse through secondary filaments or underground water channels (Cediel & Shaw, 2019), that connect between the farms selected in this study. This may create ideal conditions which, through time, may have accumulated more metals (i.e., Cd) by sedimentation processes than non-flooding areas (see Figs. 2 and 3). This kind of hypothesis could infer a potential relationship between hydrology and geology providing another possible explanation for the presence of Cd in certain landscapes of Arauca where cacao grows.

As there were significant differences in the concentration of Cd in soils within the CSUs, and good correlations with soil chemical variables such as Mg, ECEC, Zn, Ca, and SOM; it is necessary to continue studying other relationships and parameters associated with the soil composition, such as soil texture, clay minerals, mineral oxides/oxyhydroxides, properties that have shown affinity with Cd in soils according to different authors (Bravo & Braissant, 2022; Rezaei et al., 2021; Shaheen et al., 2013; Spark et al., 1995).

Soil Cd from anthropogenic sources was not considered in this study. However, factors associated with this source have been widely reported, including activities such as emission by non-ferrous metal mining and refining, fossil fuel combustion, application of phosphate fertilizers and pesticides, and waste disposal.

### Cd in soil litter

The accumulation of heavy metals in plants occurs through two processes: absorption within plant cells and translocation from the roots to other parts (Riyazuddin et al., 2021). During the translocation process, heavy metals may concentrate in greater quantities in aerial parts such as shoots and beans, mediated by the xylem (Wang et al., 2021). Some species may exhibit higher concentrations of heavy metals in older or senescent leaves, indicating an active exclusion or defence mechanism against toxic elements, including Cd (Galvis et al., 2023). Once the leaves fall off the tree, they carry the toxic element with them. A previous study (Sousa et al., 2021), conducted with rice plants, found that a high supply of NH_4_^+^ initially triggers a regulated self-destruction process induced rapidly in older leaves, followed by a high accumulation of NH_4_^+^ and death of the tissue. This mechanism prevents the flow of high amounts of the toxic element to the young leaves and other tissues, preserving photosynthetic capacity and growth when subjected to high contents of the toxic element in the root medium.

### The role of cacao varieties in the bioprocess of Cd

The variety of cacao may play a role in the bioprocess of Cd. Some studies have focused on researching that role in greenhouse experiments (Arévalo-Hernández et al., 2021) or in germplasm bank assessments where semi-controlled conditions can be found (Lewis et al., 2018). However, evaluating cacao genetic varieties in established cacao fields is a challenging task as each farm has a different genetic arrangement. Additionally, the interaction with the surrounding environment makes it a difficult factor to pursue. This was the case in this study. As shown in Table 1, each farm has a set of different genetic arrangements that are expressed ‘as an average’ by municipality.

### Cd in cacao beans and artisanal chocolate

In regards of the Cd content in cacao beans, the average content is close (0.78 mg kg^−1^) to the EU regulation (European, 2014), even though the norm was designed for final products and derivative products. Therefore, the content of this metal in cacao beans should not limit trade to Europe. Nonetheless, in some artisan chocolate bars from single farms, the Cd content exceeded the limit due to localised differences in soil Cd and other factors. This issue can be resolved by mixing beans from different farms so that the concentration in the bar is more similar to that expected from the regional cacao bean average mentioned above, suggesting to farmers, between the municipalities, to take into account the gradient of Cd from the areas close to the Arauca River to inner lands. On top of that, it is well known that 20% of Cd is lost when the husk is removed, and that there is an added reduction depending on the recipe (Bravo et al., 2022).

Cd reduction during the fermentation process has been reported with mixed findings. This could be, in part, because this topic is relatively new and not a consensus exists. For example, in one study (Zhai et al., 2019), Cd variation was attributed to the acidification during fermentation, applying lactic acid bacteria to reduce the pH below 5. In another study (Vanderschueren et al., 2020) proposes that reduction of Cd in nibs can be achieved by increasing acidification; nonetheless, they noted that extreme acidity causes changes in the flavour of chocolate, which interferes with organoleptic characteristics. In yet another study (Morales-Rodríguez et al., 2022), pectintranseliminase (PTE) was used, showing reduction in Cd content during the postharvest process. However, a simultaneous study (Bravo et al., 2022) showed that, even modifying the concentration of weak acids, such as lactic or acetic acid, or varying the days of fermentation, there was no significant different in Cd content compared with fresh seeds or unfermented beans. The results were replicated in Colombia, Peru, and Trinidad and Tobago. Therefore, more research is required to understand wheter manipulation of the cocoa fermentation processes could be viable strategy to reduce Cd concentrations.

### The role of plant material and age in Cd content in cacao

As described in Table 1, in the assessed farms in this study, four native cacao varieties from the region predominate (FSA 13, FEAR 5, FTA 2, FTA 4) across the subregions. Interestingly, some plants that uptake Cd with higher affinity are known as hyperaccumulators. This designation is given to species that meet the following three criteria: (i.) uptake and storage of 100 mg Cd per kg dry matter, (ii.) they should have a transfer factor (TF) and an internal translocation factor (ITF) greater than 1, and (iii.) exhibit extreme tolerance to heavy metals due to efficient biochemical detoxification (Kirkham, 2006; van der Ent et al., 2013; Vanderschueren et al., 2021). Cacao is a crop that uptakes Cd with affinity; however, it does not meet the first criterion (our data shows that cacao seed has a maximum of 1.19 of Cd per kg of dry matter, see Table 3), to be categorised as a hyperaccumulator (Argüello et al., 2019; Barraza et al., 2017; Gramlich et al., 2018; Lewis et al., 2018; Vanderschueren et al., 2020, 2021). Moreover, the age of the cacao crop is another key factor regarding bioaccumulation. In our study (see in Table 1 the column range of ages), we found that the youngest plantations ranging from 2.8 to 18 years were found in both the middle and south of the study area, areas with the lowest pseudototal Cd contents found in soils. At this point in time, it remains unclear if the plant-associated species in the cacao crop system drive Cd fluxes into the open systems in Arauca. Therefore, the expansion of cacao in Arauca should focus on areas that are lower in Cd, and that are becoming more suited to cacao due to climate change. Consequently, there are two take home messages. Firstly, in terms of the baseline in soils, agricultural traits of cacao, Cd is an element that has always been, and it is always going to be, in the cacao crop system. Secondly, the Cd content in cacao fresh seeds is close to the limit of the EU regulation and Cd content in artisan chocolates might be mixed between municipalities taking into account the north–south pattern of Cd assessed in Arauca.

## Conclusions

This study represents the first characterization of Cd content distribution in the local cacao chain of Arauca, Colombia. The highest Cd content was found in soil litter (10.03 mg kg^−1^ on average), while the lowest was found in cacao seeds (0.78 mg kg^−1^ on average). Furthermore, this study demonstrated that the northern municipalities of Arauca exhibited greater exposure to elevated Cd levels compared to other areas in Arauca. Interestingly, a north–south gradient in the distribution of Cd was observed due to sedimentation processes, mainly in the Arauca River. These findings could also be attributed to natural processes such as sedimentation resulting from millennial water flow trends and changes in soil types across diverse landscapes. The historical dynamics of flooding riverbanks are the main drivers explaining the cadmium content found in cacao beans at the assessed farms in Arauca, Colombia. Interestingly, Cd values in artisanal chocolates were close to the international EU regulation. Hence, a bean-mixing strategy is suggested for the municipalities studied to be able to manufacture chocolate that complies with the regulations, as a suitable short-term strategy.

### Supplementary Information

Below is the link to the electronic supplementary material.

Supplementary file1 (DOCX 113 KB)

Supplementary file2 (DOCX 464 KB)

Supplementary file3 (DOCX 153 KB)

Supplementary file4 (DOCX 60 KB)

## Data Availability

Since Cd data in cacao is an economic and social issue in Colombia, the data are not public; however, part of the data could be considered to share after contacting officially the corresponding author and requesting it to Agrosavia.
