# Supplementary material for: Assessing the cadmium content of cacao crops in Arauca, Colombia
Source: Environ Monit Assess. 2024 Mar 21;196(4):387. doi: 10.1007/s10661-024-12539-9 (PMC10954870; doi:10.1007/s10661-024-12539-9)
Supplement: Supplementary file 1 — Supplementary file1 (DOCX 113 KB) [file 10661_2024_12539_MOESM1_ESM.docx]

Assessing the cadmium content of cacao crops in Arauca, Colombia

Authors: Daniel Bravo^1*^, Ruth Quiroga-Mateus^1^, Marcela López-Casallas^2^, Shirley Torres^2^, Ramiro Contreras^2^, Andres Camilo Mendez Otero^2^, Gustavo A. Araujo-Carrillo^3^, Carlos E. González-Orozco^2^

Supplementary material Figure S1.


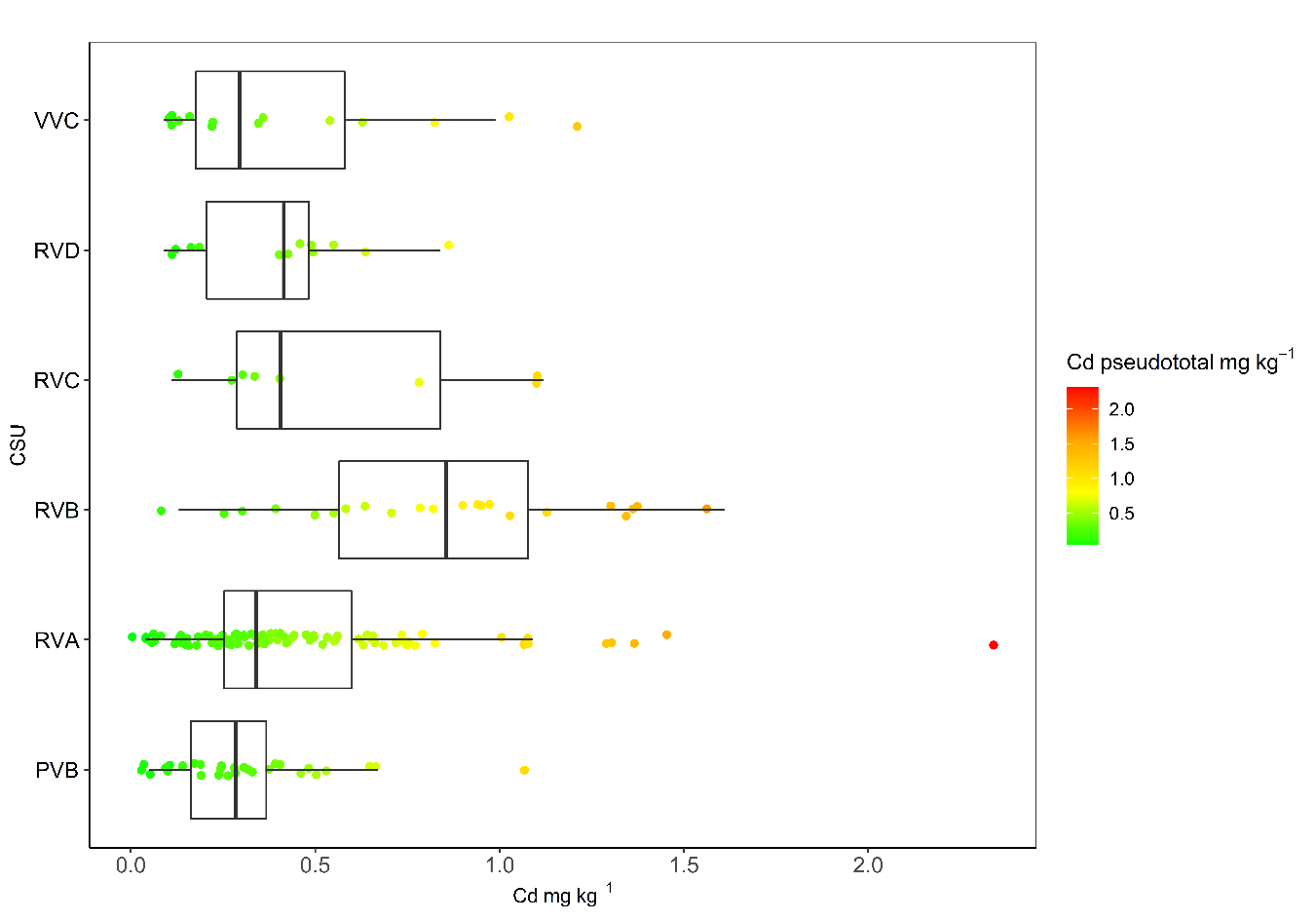


**Figure S1.** Boxplots of pseudo-total Cd in soils regarding Cartographic Soil Units (CSU) from 180 assessed farms in Arauca.
